# Growth Differentiation Factor 15 Ameliorates Anti-Glomerular Basement Membrane Glomerulonephritis in Mice

**DOI:** 10.3390/ijms21196978

**Published:** 2020-09-23

**Authors:** Foteini Moschovaki-Filippidou, Stefanie Steiger, Georg Lorenz, Christoph Schmaderer, Andrea Ribeiro, Ekaterina von Rauchhaupt, Clemens D. Cohen, Hans-Joachim Anders, Maja Lindenmeyer, Maciej Lech

**Affiliations:** 1 LMU Klinikum, Medizinische Klinik und Poliklinik IV, Ludwig-Maximilians-Universität Munich, 80336 München, Germany; foteini.moschovaki@gmail.com (F.M.-F.); stefanie.steiger@med.uni-muenchen.de (S.S.); andrea.sof@gmail.com (A.R.); katjats95@gmail.com (E.v.R.); C.Cohen@med.uni-muenchen.de (C.D.C.); hjanders@med.uni-muenchen.de (H.-J.A.); 2 Klinikum rechts der Isar, Department of Nephrology, Technical University Munich, 81675 München, Germany; georg.lorenz.gl@googlemail.com (G.L.); christoph.schmaderer@mri.tum.de (C.S.); 3 III. Department of Medicine, University Medical Center Hamburg-Eppendorf, 20246 Hamburg, Germany; m.lindenmeyer@uke.de

**Keywords:** inflammation, T cells, glomerulonephritis, innate immunity, chemokines

## Abstract

Growth differentiation factor 15 (GDF15) is a member of the transforming growth factor-β (TGF-β) cytokine family and an inflammation-associated protein. Here, we investigated the role of GDF15 in murine anti-glomerular basement membrane (GBM) glomerulonephritis. Glomerulonephritis induction in mice induced systemic expression of GDF15. Moreover, we demonstrate the protective effects for GDF15, as GDF15-deficient mice exhibited increased proteinuria with an aggravated crescent formation and mesangial expansion in anti-GBM nephritis. Herein, GDF15 was required for the regulation of T-cell chemotactic chemokines in the kidney. In addition, we found the upregulation of the CXCR3 receptor in activated T-cells in GDF15-deficient mice. These data indicate that CXCL10/CXCR3-dependent-signaling promotes the infiltration of T cells into the organ during acute inflammation controlled by GDF15. Together, these results reveal a novel mechanism limiting the migration of lymphocytes to the site of inflammation during glomerulonephritis.

## 1. Introduction

Anti-glomerular basement membrane glomerulonephritis (anti-GBM nephritis) is a severe acute kidney disease characterized by a variety of lesion patterns, including crescents, vascular loop necrosis, and mesangial expansion, suggesting multiple pathogenic mechanisms [1,2,3,4]. The upstream role of the adaptive immune system in failing to maintain immune tolerance and its downstream role in causing antigen-specific immunopathology in the glomerular compartment of the kidney has been widely recognized [3,5,6,7]. Experimental and clinical studies confirmed that various pro-inflammatory mediators are involved in this process [8,9,10]. For example, chemokines and transforming growth factor-β (TGF-β) family members regulate the inflammatory process and determine the progression and outcome of anti-GBM nephritis [11,12,13,14].

Growth differentiation factor 15 (GDF15, also known as macrophage inhibitory cytokine-1, MIC-1) is produced as a 35 kDa full-length form, cleaved in the N-terminus and secreted as a 25 kDa mature form [15]. GDF15 is a divergent member of the TGF- β superfamily and shows several structural differences compared to the rest of the superfamily. GDF15 is a regulator of inflammatory response dendritic cells maturation [16] and peripheral blood mononuclear proliferation [17]. Moreover, its role was suggested in cervical cancer, glioblastoma, cardiovascular ischemic stress, obesity, and metabolic disease, such as mitochondrial myopathies [18,19,20,21,22,23,24]. However, the knowledge of its role in progression of glomerulonephritis (GN) and mechanisms of function is still limited and requires further investigation. T cell-driven effector mechanisms play an important role, particularly in crescentic GN [25], and understanding the function of T-cells in the disease can be beneficial in future therapies for glomerular injury. Some studies showed a functional role for CD4+ T cells as effector cells participating in the development of crescentic GN by executing delayed-type hypersensitivity [26]. Others identified CD4+ cells but not CD8+ cells as crucial for the development of crescentic GN in mice [27]. By contrast, some findings suggest that glomerular injury in anti-GBM GN is driven by macrophage recruitment, which depends on both CD4+ and CD8+ T cells, and that T cell cytotoxicity does not play a role in the progression of the disease [28]. However, an anti-CD8 monoclonal antibody therapy was effective in both the prevention and treatment of experimental autoimmune glomerulonephritis [29], which could be associated with the effects of CD8+ cells on ICAM-1 and cytokine expression in crescentic glomerulonephritis [30]. Until now, systemic GDF15 levels have been shown to correlate with the progression of chronic kidney disease (CKD). Recombinant GDF15 protein reduced fibroblast activation and interstitial fibrosis in a model of unilateral ureter obstruction (UUO), possibly by blocking the TGF-β receptor and N-Myc signaling pathways [31]. Moreover, GDF15 reduces the expression of MHC class II and co-stimulatory molecules, as well as NF-kB family members Rel A and Rel B, IL-2, IFN-γ, and IL-12p40, and increases expression of TGF-β, and IL-10 [32]. In general, GDF15 is believed to be associated with stress responses, but its precise biological functions remain to be elucidated. Although GDF15 has been shown to affect aspects of chronic diseases (both inflammation and fibrosis), whether it controls glomerular disease progression is unclear [31,33,34].

In the present study, we used GDF15-deficient mice to investigate the role of GDF15 in anti-GBM nephritis. We hypothesized that GDF15 ameliorates the anti-Glomerular Basement Membrane (GBM) glomerulonephritis by limiting the inflammation and infiltration of the immune cells into the kidney.

## 2. Results

### 2.1. Experimental Anti-GBM Nephritis Is Associated with Higher Systemic and Intrarenal Expression of GDF15

**Figure 1:**
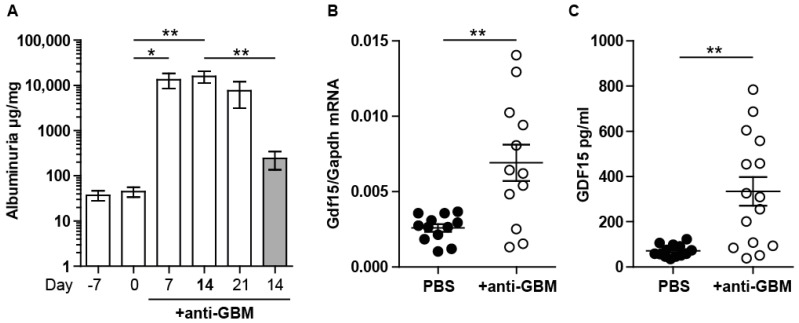
Evaluation of the anti-GBM model and expression of GDF15. (**A**) We used the commercially available GBM antiserum that was raised in sheep against rat GBM. We first examined its nephritogenic potential in C57BL/6 mice by assessing albuminuria 7, 14, and 21 days after a single intravenous injection of antiserum in pre-immunized mice, as well as in mice without pre-immunization (gray bar, 14 days). (*n* = 5, one-way ANOVA). (**B**) Total RNA isolated from kidneys of saline- or antiserum-injected C57BL/6 mice underwent quantitative real-time RT-PCR analysis and revealed significantly higher expression of Gdf15 in treated mice. (**C**) Serum GDF15 level was significantly increased in antiserum-injected C57BL/6 mice (*n* = 12, Student’s *t*-Test). Data are mean ± SEM. * *p* < 0.05; ** *p* < 0.01.

We first asked if we could detect increased levels of GDF15 in mice following anti-GBM nephritis. To develop a reliable model, we tested several protocols and chose one with significant proteinuria and histopathological changes (Figure 1A). The nephrotoxic potential of the GBM antiserum in C57BL/6 mice (without pre-immunization) was previously assessed [35]. To implement the autologous adaptive immune response, we immunized mice with sheep-IgG 7 days prior to anti-GBM serum injection. We compared the development of albuminuria from day 0 to 21 in order to define the time-point with stable glomerular injury (Figure 1A). Moreover, we compared the amount of albuminuria of pre-immunized mice with mice without pre-immunization (heterologous model). Pre-immunization and the administration of anti-GBM serum resulted in a significant increase in albuminuria at 7 and 14 days. RT-PCR analysis of *Gdf15* and serum analysis of the protein revealed a low basal expression level, which was significantly upregulated 14 days upon anti-GBM serum injection (Figure 1B,C). We conclude that GDF15 is induced anti-GBM nephritis and that our protocol (7 + 14 days) of autologous anti-GBM nephritis is suitable to study the role of GDF15 in glomerular inflammation.

### 2.2. GDF15 Deficiency Aggravates Albuminuria, Kidney Function Loss, and More Severe Tubular and Glomerular Injury in Anti-GBM Nephritis

**Figure 2:**
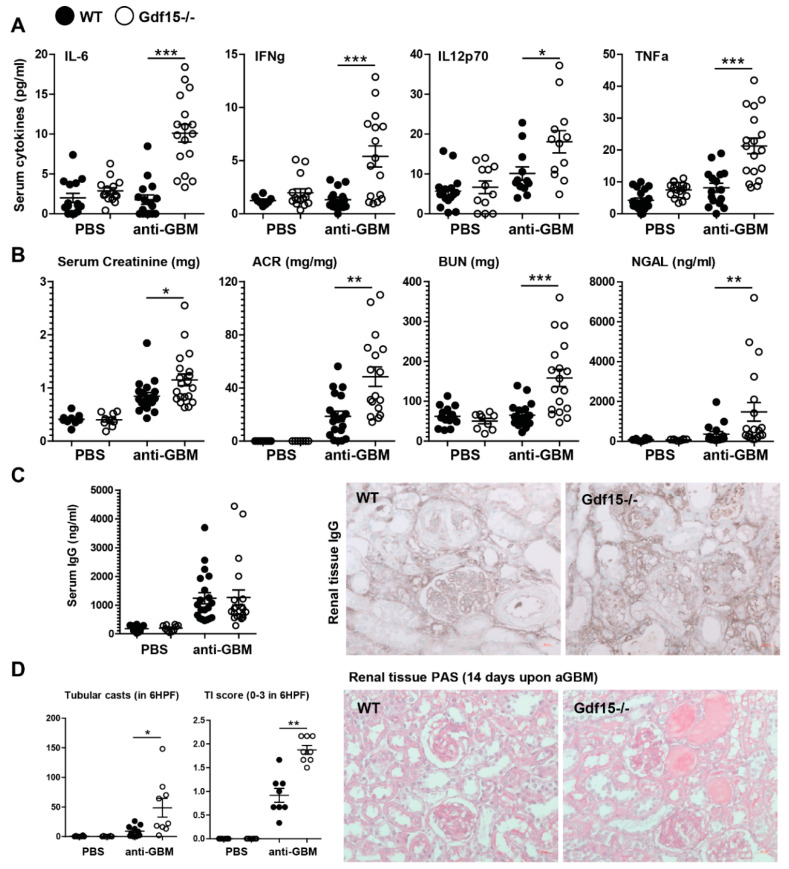
Systemic inflammation, kidney function, and histopathology of anti-GBM nephritis. (**A**) Sera were obtained from wild type or GDF15-deficient C57BL/6 mice on day 14 after saline or antiserum (anti-GBM) injection. Cytokine levels were quantified by flow cytometry (*n* = 15–17, one-way ANOVA). (**B**) Renal function parameter (*n* = 15–17, one-way ANOVA). (**C**) Serum IgG levels (*n* = 15–17, one-way ANOVA) and immunohistochemistry staining for IgG on kidney sections were quantified. (**D**) Kidneys from WT or KO mice were paraffin-embedded, stained with Periodic acid-Schiff (PAS) reagent, and quantified to assess tubular casts formation and tubular injury score (*n* = 8 mice per group, one-way ANOVA). Representative images of renal sections (original magnification 400×). Data are mean ± SEM. * *p* < 0.05; ** *p* < 0.01; *** *p* < 0.001.

In order to address the role of GDF15 in glomerular inflammation, we applied the same protocol to C57BL/6 mice and *Gdf15*-/- mice. Plasma levels of multiple cytokines revealed an increased pro-inflammatory systemic signature. We detected significantly increased levels of IL-6, IL-12, TNF-α, and IFN-γ in the serum of GDF15-deficient mice (Figure 2A). Significantly increased glomerular filtration markers such as serum creatinine and blood urea nitrogen, glomerular injury markers-i.e., albuminuria (albumin/urine creatinine ratio), and tubular injury markers-i.e., urinary lipocalin-2 (NGAL) were noted in *Gdf15*-/- mice compared with wild type controls, which indicates more severe kidney disease in knockout mice (Figure 2B).

Based on these data, we assumed that GDF15 might play a protective role in anti-GBM nephritis. Both ongoing inflammation and severe glomerular injury can cause tubular injury. As expected, kidney sections stained with Periodic acid Schiff (PAS) reagent revealed increased tubular cast formation and tubular atrophy (scored as tubular injury TI) in nephritic GDF15-deficient animals compared to nephritic wild type animals (Figure 2D). These results demonstrate that the systemic deletion of *Gdf15* ameliorates proteinuria and renal tubular injury in anti-GBM nephritis. We did not observe any significant differences in total IgG levels in the blood of wild type and knockout mice. Consequently, the whole IgG staining of renal tissue did not reveal significant differences between the two treated groups (Figure 2C).

**Figure 3:**
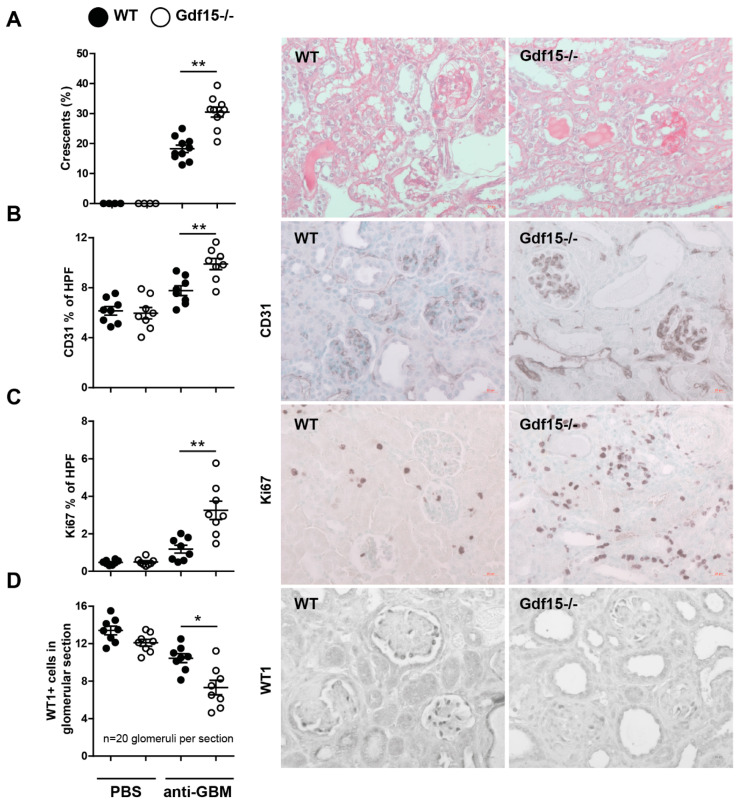
Kidney histopathology of anti-GBM nephritis. Sections were obtained from wild type or GDF15-deficient C57BL/6 mice on day 14 after saline or antiserum (anti-GBM) injection. Kidneys from WT or KO mice were paraffin-embedded and stained either with (**A**) PAS, (**B**) anti-CD31 antibody, (**C**) anti-ki67 antibody and (**D**) anti-WT1 antibody, and quantified (*n* = 8 mice per group, one-way ANOVA). Representative images of renal sections (original magnification 400×). Data are mean ± SEM. * *p* < 0.05; ** *p* < 0.01.

Because the majority of patients with an anti-GBM disease develop widespread glomerular crescent formation followed by features of rapidly progressive glomerulonephritis, we quantified the number of glomerular crescents of *n* = 8 mice per group. We showed that GDF15-deficient mice displayed enhanced crescent formation (Figure 3A). As endothelial cells (ECs) are involved in the inflammatory process in glomeruli and the progression of glomerulonephritis, we investigated by immunohistochemistry the expression of CD31. Glomerular endothelial injury leads to podocyte loss and proteinuria. A cross-sectional evaluation revealed that the glomeruli of *Gdf15*-/- mice strongly expressed the CD31 marker (Figure 3B). Importantly, we observed the differences in glomerular architecture between wild type and knockout mice, indicating distinct stages of glomerulonephritis. To assess the cellular (including endothelial cell) proliferative responses, we stained renal sections with the antibody against Ki67. We observed increased proliferation of the cells in glomeruli as in the other renal compartments. This indicates the higher rearrangement (triggered by higher injury) of the tissue of knockout mice compared to wild types (Figure 3C). Furthermore, *Gdf15*-/- mice with anti-GBM nephritis exhibited a decreased number of WT1 positive podocytes in the glomeruli (Figure 3D). These pathological features explained massive albuminuria, increased BUN, and serum creatinine observed (Figure 2B). Together, lack of GDF15 induces severe renal disease upon anti-GBM serum treatment, whereas control mice (WT anti-GBM) displayed a less severe phenotype. These data emphasize an indispensable role for GDF15 in kidney disease.

### 2.3. Gdf15-Deficient Mice Exhibit Increased Renal Inflammation in Anti-GBM Nephritis Model

**Figure 4:**
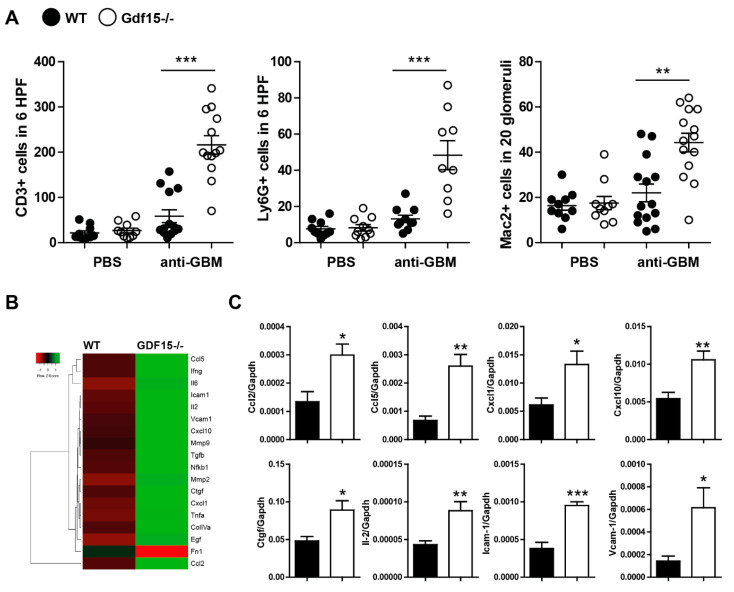
Kidney inflammation in wild type and *Gdf15* KO mice with anti-GBM nephritis. (**A**) Kidney sections were stained with anti- CD3, Ly6G, or Mac2 antibodies and quantified by counting, as indicated on graphs and in material and methods (*n* = 9–15 mice per group, one-way ANOVA). (**B**) Heat map depicting kidney expression of pre-selected genes of wild type and GDF15-deficient mice upon anti-GBM serum treatment. (**C**) Gene expression levels in kidneys were quantified by real-time PCR. Data are shown as means of the ratio of the specific mRNA vs. that of *Gapdh* mRNA (*n* = 6–8 samples per group, Student’s *t*-Test). Data are mean ± SEM. * *p* < 0.05; ** *p* < 0.01; *** *p* < 0.001 versus control mice.

Further, we investigated the impact of GDF15 on renal inflammation as one of the key determinants of glomerular damage and albuminuria in the early phase of anti-GBM nephritis. We hypothesized that the mechanism underlying severe glomerulonephritis in *Gdf15*-/- mice could be associated with T cells. T-cells play a crucial role in proliferative and crescentic glomerulonephritis and can be a trigger of severe and rapid damage even in the absence of glomerular antibody deposition [3,8,36]. Indeed, we observed a significant increase in the number of CD3+ T cells, neutrophils, and macrophages on kidney sections from anti-GBM-treated *Gdf15*-/- mice compared with WT mice (Figure 4A). At the preselected mRNA level, the expression of the glomerular extracellular matrix proteins, matrix remodeling enzymes matrix metalloproteinase-7 and matrix metalloproteinase-9, as well as inflammation markers, was higher in anti-GBM-treated *Gdf15*-/- mice compared with WT mice on day 14 (Figure 4B). However, the further quantification of samples revealed that the expression of chemokines/cytokines *Ccl2, Ccl5, Cxcl1, Cxcl10, Ctgf, and Il2*, as well as adhesion molecules *Icam1* and *Vcam1*, were significantly more expressed in GDF15-deficient mice compared to wild type mice upon anti-GBM serum treatment. Together, the systemic lack of GDF15 leads to an increased progression of renal disease by orchestrating inflammation and immune cell influx.

### 2.4. T Cells from Wild Type and Knockout Mice Display No Differences in Expression of Adhesions Markers

**Figure 5:**
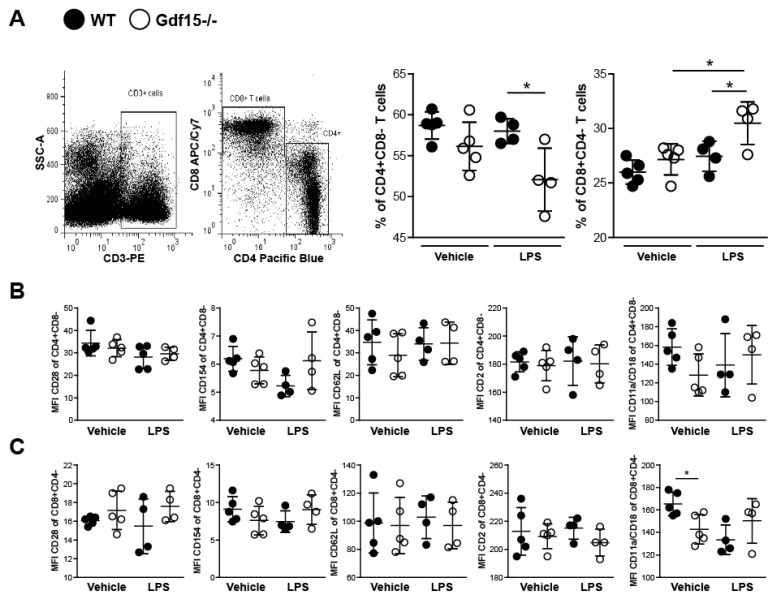
Stimulation of splenic T cells from WT and *Gdf15* KO mice with or without LPS ex vivo. (**A**) T cells were isolated from WT and *Gdf15* KO mice and stimulated with or without LPS for 24 h. The percentage of CD4+CD8- and CD8+CD4- T cells was quantified by flow cytometry (gating strategy, *n* = 4–5 per group, one-way ANOVA). (**B**,**C**) Mean fluorescence intensity (MFI) of the surface markers CD28, CD154, CD62L, CD2, and CD11a/CD18 on LPS-stimulated or untreated CD4+CD8- T cells (**B**) and CD8+CD4- T cells (**C**) (*n* = 4–5 per group, one-way ANOVA). Data are mean ± SD. * *p* < 0.05.

Due to the observed kidney influx of CD3+ cells and the increased IL-2 expression (crucial T-cell cytokine), we hypothesized that T cells from *Gdf15*-/- mice could also have an adhesion associated phenotype. To evaluate the effect of GDF15-deficiency on the adhesive capacity of T cells, we established an in vitro system in order to analyze the expression of several adhesion markers on T cells isolated from spleens. After the activation of naïve T cells with LPS, we did not observe increased expression of CD28, CD154, CD62L, and CD11a/CD18 in CD4+ T cells (Figure 5B) nor CD8+ T cells (Figure 5C) in comparison with unprimed vehicle-treated cells. We did not see any significant differences between WT and KO cells. After in vitro stimulation, T cells from wild type and *Gdf15*-/- mice showed no differences in TCR internalization, evidenced by the reduction in CD3 MFI (data not shown). Interestingly, we observed a shift towards CD8+ cytotoxic T cells in primed GDF15-deficient cells (Figure 5A). The accumulation of effector CD8+ T lymphocytes may suggest increased damage to the surrounding tissue.

**Figure 6:**
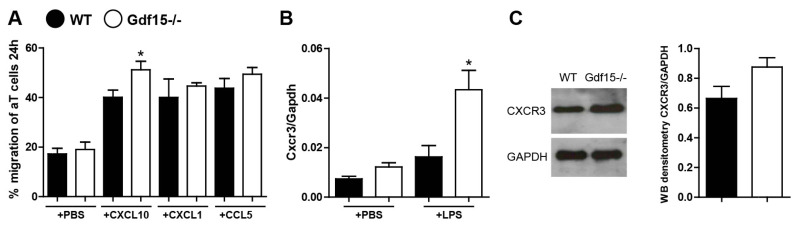
Quantification of T cell migration and CXCR3 expression. (**A**) T cells from the spleens of WT and GDF15-deficient mice were isolated and migration assays were performed. The percentage of migrated T cells towards the chemokines CXCL10, CXCL1, and CCL5 was quantified after 24 h (*n* = 4 per group, one-way ANOVA, * *p* < 0.05). (**B**) Cxcr3 expression levels in LPS-stimulated or untreated T cells were quantified by real-time PCR. Data are shown as means of the ratio of the Cxcr3 mRNA vs. Gapdh mRNA (*n* = 4 per group, one-way ANOVA, * *p* < 0.05). (**C**) CXCR3 protein expression levels in LPS-activated T cells were quantified by Western blot. The histogram shows the densitometry of two independent experiments; no statistical analysis was performed. Data are mean ± SEM.

As the trafficking of T cells to tissues mainly depends on the tissue microenvironment, we decided to investigate the effect of chemokines that were highly expressed during anti-GBM nephritis on the migratory potential of T cells. We used both naïve and activated T cells (interaction with APC upon LPS treatment), and analyzed their migration towards the chemokines CXCL1, CXCL10, and CCL5 using migration chambers with micro-channels for 24 h. We observed a significant increase in the percentage of T cells from GDF15-deficient mice that migrated towards CXCL10 but not CXCL1 and CCL5 (Figure 6A). CXCL10 is known to be highly expressed during kidney disease [37,38,39]. Its receptor CXCR3 is expressed on effector T cells and crucial in T cell trafficking and function [40,41]. Moreover, IFNγ activates the Stat-dependent pathway in tissue-resident cells to enhance the production of CXCL10 during tissue injury. We previously observed high Cxcl10 expression in kidney tissue and high systemic levels of IFN-γ in treated *Gdf15* KO mice. We hypothesized that the elevated number of effector T cells might not only be associated with the CXCR3 receptor. Therefore, we looked at the level of Cxcr3 in naïve and activated T cells. Indeed, we found a significantly higher mRNA and protein levels of CXCR3 in T cells from GDF15-deficient mice upon activation (Figure 6B,C). Taken together, these data indicate that GDF15 plays a role in the migration of T cells to the side of inflammation via a CXCL10-CXCR3 dependent mechanism.

### 2.5. Gene Expression Analysis of GDF15, CXCL10 and CXCR3 in Patients with RPGN

**Figure 7:**
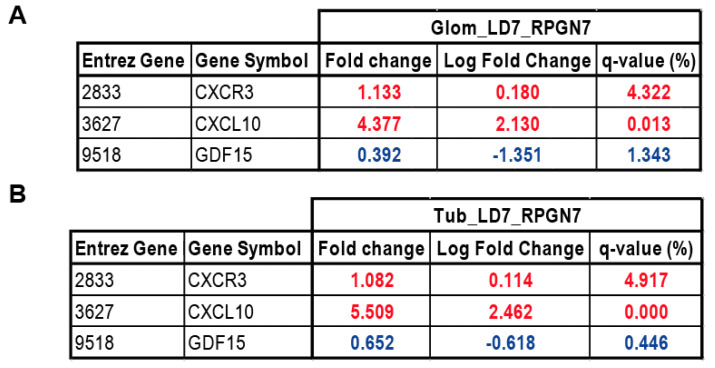
Gene expression analysis of *GDF15, CXCL10*, and *CXCR3* genes in (**A**) glomerular and (**B**) tubular compartment of manually microdissected kidney biopsies from patients with RPGN. Values are expressed as a log2-fold change compared to controls (living donors, LD). All represented genes are significantly changed (*q* < 0.05). (**A**) Glomerular expression single hybridization (LD: *n* = 18, RPGN: *n* = 23), (**B**) Tubular compartment expression (LD: *n* = 18, RPGN: *n* = 21). Red represents upregulation and blue represents downregulation of the transcript.

In order to estimate the transferability of our results to human disease, we assessed transcriptional levels of *GDF15, CXCL10,* and *CXCR3* in the glomerular and tubular compartment from human renal biopsy specimens using Affymetrix microarray expression data. For this project, we used biopsies of individuals with rapidly progressive glomerulonephritis (RPGN) versus living donor (LD) biopsy specimens (Figure 7). In both, the glomerular and the tubular compartment of the patient cohort, *GDF15* was significantly downregulated in patients with RPGN compared with controls. At the same time, the *CXCL10* and its receptor *CXCR3* were significantly upregulated it both the glomerular and tubular compartment. Together, genes that seem to be regulated by GDF15 in the experimental mouse model of anti-GBM were induced during rapidly progressive glomerulonephritis in the glomerular and tubular compartment of the kidney.

## 3. Discussion

We hypothesized that a GDF-15-associated mechanism is responsible for the development of GBM. Our results now show that the cytokine GDF15 protects from kidney injury and inflammation in a mouse model of anti-GBM nephritis. Furthermore, we demonstrate that Gdf15 is induced locally as well as systemically in the injured kidney and that GDF15 acts as an anti-inflammatory cytokine by regulating systemic cytokine production. GDF15 is involved in the recruitment of T cells into the injured tissue, a phenomenon associated with CXCL10-CXCR3 signaling. This finding is in line with previous reports on the functional role of GDF15 demonstrating that this cytokine can regulate chemokine-triggered β2 integrin activation on myeloid cells [42]. We further expand this knowledge by reporting on the enhanced migratory capability of peripheral blood mononuclear cells towards CXCL10 ex vivo and provide evidence for increased numbers of infiltrating immune cells in kidneys of mice with anti-GBM nephritis. The accumulation of T lymphocytes within the kidney might display a key event contributing to kidney injury. Some of the studies evidenced that anti-GBM GN depends on both CD4 and CD8 T cells without direct T cell-mediated cytotoxicity [28]. Other investigations support the hypothesis that especially CD8+ T cells are important mediators of glomerulonephritis [43]. Nevertheless, chemokines and chemokine receptors play a role in the process of T cell recruitment to the site of inflammation. Here, we show that both CXCL10, as well as its receptor, might be regulated by GDF15. Previous studies highlighted the importance of CXCL10 during inflammation and showed that CXCL10 is critical for the recruitment of CXCR3-expressing effector T cells to the injured tissue [44]. In in vivo models, CXCR3-expressing CD4+ and CD8+ T cells did not migrate efficiently into inflamed tissues of CXCL10-deficient mice compared to wild type, suggesting that CXCR3 expression is crucial for the selection of cells that respond to CXCL10 [44]. Consistent with earlier studies, our data indicate that GDF15 limits the inflammatory response during injury by restricting immune cell infiltration. Kempf et al. showed that GDF15 protects against fatal cardiac rupture by counteracting chemokine-triggered β2 integrin activation on myeloid cells [42]. This, for the first time, implied that GDF15 might be involved in the regulation of cytoskeleton rearrangement. In our experiments, except for changes in inflammation and significant effects of GDF15 on chemokine production, we could observe significant upregulation of *Icam* and *Vcam* adhesion molecules in the injured kidneys of *Gdf15*-/- mice. This suggests pleiotropic effects of GDF15 in the process of progressive GN, whereby GDF15 orchestrates the migration of T cell populations into the kidney. Therefore, treatment with GDF15 seems to be more beneficial than blocking particular chemokines and chemokine receptors to inhibit the selective migration of defined cell populations to the site of inflammation.

Other mechanisms potentially contributing to exaggerated injury in GDF-15-deficient mice include the polarization of cytotoxic CD8 T cells. Although overall surface marker expression upon LPS stimulation was comparable to wild type, we observed a significant decrease in the number of CD4+ T cells but an increase in CD8+ T cells upon activation in knockout cells. This suggests a distinct immune response associated with specific T cell subsets and more severe clinical manifestations in GDF15-deficient mice. CD8+ cytotoxic T cells serve as a potent source of inflammation by producing perforin and granzymes and inducing apoptosis in target cells [45,46,47]. By contrast, CD4+ helper T cells support the immune responses by promoting the activation and/or proliferation of immune cells [48]. The polarization of T cells towards CD8+ T cells could be an additional explanation for the severe phenotype observed in knockout mice.

In addition, we observed a protective role of GDF15 in both the glomerular and tubular compartment as evidenced by increased macrophage infiltration and expression of inflammatory markers in *Gdf15*-/- mice, indicating that glomerular inflammation in autologous anti-GBM disease involves both innate and adaptive immunity [49,50] and that GDF15 could act as a regulator of both immune responses. Currently, it is unclear whether the injured glomeruli cause tubular damage or whether both processes occur independently of each other due to the accumulation of immunoglobulins and immune complexes in the kidney. In our model, the glomerular damage is accompanied by tubule interstitial damage and both seem to depend on GDF15, because the injury in knockout mice is significantly higher. Therefore, we investigated both the tubular and glomerular compartment of patients with RPGN. RPGN is associated with a high frequency of crescentic glomerulonephritis [51]. Since we observed significant changes in GDF15 in an experimental anti-GBM model, as well as enhanced CXCL10-CXCR3 signaling, we chose to compare the expression of these three transcripts. Our analysis revealed the significant downregulation of GDF15 and at the same time the upregulation of CXCL10 and CXCR3. In support of our experimental findings, the human data suggest that the level of GDF15 affects the infiltration of CXCR3-expressing T cells to the site of injury. For instance, Dai et al. showed that the anti-CXCR3 treatment of mice inhibits autoreactive CD8+ T cells in the skin and peripheral lymphoid tissues [52]. Moreover, in a mouse model of vitiligo, CXCL10-/- unlike CXCL9-/- mice did not develop depigmentation, indicating that the CXCL10-CXCR3 axis is responsible for this disease [53]. In humans, the blockade of CXCL10 showed positive effects on the progression of rheumatoid arthritis, indicating a therapeutic approach in blocking the CXCL10-CXCR3 axis not only in experimental mouse models but also in human disease [54].

Stable and accurate noninvasive markers, as well as a better understanding of the pathophysiology of rapidly progressive GN could enable fast, precise diagnosis and appropriate treatment of the disease. Until now, several studies showed that GDF15 is associated with a rapid decline in kidney function, suggesting that might be a useful predictor for the development of kidney disease. For instance, recent investigations of two independent cohorts showed that systemic GDF15 levels correlate with the intrarenal expression of *GDF15* and are significantly associated with the progression of kidney disease [55]. Enhanced GDF15 levels correlate not only with increased mortality in hemodialysis and diabetic nephropathy (DN) patients [56,57] but also with all-cause mortality [58,59]. Moreover, in DN patients, high levels of GDF15 are associated with a decline in estimated glomerular filtration rate and faster progression to kidney failure [57]. Surely, the enhanced levels of GDF15 can deliver information about the deterioration of kidney function [60,61,62]. However, the suitability of GDF15 as a precise marker for kidney disease needs further investigation. Moreover, its role as a urinary marker on the progression of kidney disease, as well as the correlation of plasma and urine GDF15 levels, needs to be elucidated [63]. Our experimental study with GDF15-deficient mice proves the strong impact of the protein on the outcome of kidney disease and shows increased systemic and kidney expression of GDF15 during experimental GN. This increase in GDF15 levels during kidney diseases is associated with the increased production of GDF15 in response to stress (unpublished data) rather than with decreased protein clearance from circulation. Collectively, our data suggest that GDF15 reduces the outcome of experimental GN and contributes to pathogenesis by modulating T cell phenotypes and reducing the accumulation of T cells in the kidney. GDF15 may serve as a useful therapeutic molecule that could reduce disease progression by its effect on CXCL10 and CXCR3. The wide range of reported effects is diverse and the inconsistency of the published functions can be referred to by the commercial source of recombinant GDF15, which was contaminated with bioactive concentrations of TGF-β1 [64]. To avoid this issue, we used mice deficient in the GDF15 protein, which consistently proves the immunomodulatory functions of GDF15 in glomerular disease.

## 4. Materials and Methods

Animal studies: GDF15-deficient mouse strain (MGI: 2386300, *Gdf15tm1Sjl*) were backcrossed to the C57BL/6 strain. Female and male mice were housed in sterile filter top cages with a 12 h dark/light cycle. The study was carried out following the principles of the Directive 2010/63/EU on the Protection of Animals Used for Scientific Purpose and with approval by the local government authorities (27.02.2015; ROB 55.2-1-54-2532-63-12). Induction of anti-GBM nephritis: For the induction of anti-GBM nephritis, mice were pre-immunized subcutaneously with 100 μL of 2 mg/mL sheep IgG (Jackson ImmunoResearch Laboratories Inc., West Grove, PA, USA), dissolved in complete Freund’s adjuvant (Sigma-Aldrich, St. Louis, MI, USA). Five days later, sheep anti-rat glomeruli (GBM) serum (Probetex, San Antonio, TX, USA) was injected via the tail vein.

Kidney parameters: Urinary albumin excretion was evaluated by a double-sandwich ELISA. First, 96-well plates were coated with goat anti-mouse albumin antibody A90-13A-5 (Bethyl Laboratories, Montgomery, TX, USA) and plates were incubated overnight at 4 °C. After blocking for half an hour at room temperature in 0.5% BSA in PBS with 0.05% Tween20, urine samples, and mouse albumin standard (Sigma-Aldrich, St. Louis, MI, USA) were added on the plate in triplicates for 2 h. Mouse urine samples were diluted in serial dilutions ranging from 1:10^2^ to 1:10^7^. As a secondary antibody, HRP-conjugated anti-mouse albumin antibody A90-134P-7 (Bethyl Laboratories) was used. Urinary Lipocalin-2/NGAL levels were determined using a commercially available mouse ELISA DuoSet kit (R&D Systems, Park Abingdon, UK), according to manufacturer’s recommendations. The samples and standard curve dilutions were added on the plate in triplicates and incubated for two hours. Mouse urine samples were diluted in serial dilutions ranging from 1:10^3^ to 1:10^6^. TMB substrate solution (BD, Franklin Lakes, NJ, USA) was applied and the reaction was stopped after fifteen minutes with 2 M H_2_SO_4_. OD was measured at 450 nm and the calculation of the final concentrations was performed using a four-parameter logistic curve. Creatinine levels in urine and serum were determined using the creatinine assay DiaSys kit (Diagnostic Systems GmbH, Holzheim, Germany). Urine samples were prepared in dilutions of 1:10, while serum was used undiluted. According to the manufacturer’s recommendations, 10 μL of sample or standard was pipetted on 96-well plates in triplicates. Then, 200 μL of prepared reagent provided by the kit was added and absorbance was measure at 492 nm 60 s later. Two more measurements were performed 120 s and 20 min later. Concentrations were calculated using a linear standard curve. Kidney function was determined by measuring serum blood urea nitrogen (DiaSys Diagnostic Systems) and serum creatinine levels determined by the Jaffe method (DiaSys Diagnostic Systems).

Evaluation of kidney histopathology: Organs were fixed in 4% buffered formalin and embedded in paraffin. For quantitative analysis, cells were counted in sections (from at least 8–16 mice per group) or analyzed using Adobe Photoshop CS4Extended (% of stained high power field). The PAS score evaluation was performed following a semi-quantitative scoring system with a scale from 0 to 3. Samples were blinded before evaluation. CD3 positive and Ly6G positive cell quantification was performed by counting the number of positive cells in six adjacent high-power fields (Hpf) of the renal cortex and medulla. For the evaluation of the Mac-2 staining, stained cells in 20 glomeruli per sample were counted.

Mouse IgG detection: The levels of mouse IgG in the serum were determined using the mouse IgG ELISA kit (Bethyl Laboratories), according to the manufacturer’s recommendations. Briefly, 96-well plates were coated with coating antibody provided by the kit diluted (1:100) in 0.05 M Carbonate Bicarbonate with pH = 9.6 overnight at 4 °C. After 30 min blocking in 1% BSA in TrisNaCl samples and standard were added on the plate and incubated for one hour. Following five washing steps with TrisNaCl, the detection antibody was added on the plate in a dilution of 1:50,000. After the washing steps, TMB substrate solution (BD) was applied and the reaction was stopped with 2 M H_2_SO_4_. OD was measured at 450 nm and the calculation of the final concentrations was performed using a four-parameter logistic curve.

Flow cytometry: T cells were isolated from spleens of WT and *Gdf15*-/- mice and stimulated with or without LPS ex vivo for 24 h. After stimulation, T cells were collected, centrifuged, and resuspended in wash buffer (0.1% BSA, 0.01% sodium azide in D-PBS). After blocking the Fc receptor with anti-mouse CD16/32 (2.4G2) for 5 min, cells were stained with the surface antibodies FITC anti-mouse CD28, PacificBlue anti-mouse CD4, APC/Cy7 anti-mouse CD8a, PE anti-mouse CD3, PE/Cy7 anti-mouse CD154, PerCP/Cy5.5 anti-mouse CD2, APC anti-mouse CD11a/CD18, BV510 anti-mouse CD62L (all from BioLegend) for 30 min. After staining, cells were washed with wash buffer and flow cytometry was performed using the BD FACS Canto II (Becton Dickinson, New Jersey, USA). Data were analyzed with the software FlowJo 8.7 (Tree Star Inc., Ashland, OR, USA). For cytokine analysis of serum from mice or cell culture experiments, samples were prepared according to the instruction of the BD Cytometric Bead Array Mouse Inflammation Kit. The concentrations of the cytokines IL-6, IL-10, MCP-1, IFN-γ, TNF, and IL-12p70 in the samples were determined by the software FlowJo 8.7.

Real-time quantitative PCR: SYBR Green Dye detection system was used for quantitative real-time PCR on Light Cycler 480 (Roche, Mannheim, Germany). Gene-specific primers (225 nM, Metabion, Martinsried, Germany) were used. Standard controls for genomic DNA contamination, RNA quality, and general PCR performance were included. RNA was isolated from the samples using the Norgen Biotek Total RNA Purification kit (Thorold, ON, Canada) and MagNA Lyser Green beads (Roche, Basel, Switzerland) according to the manufacturer’s instructions. The data were evaluated using the 2ΔΔCT method.

Patients and microarray analysis: Human kidney biopsy specimens and Affymetrix microarray expression data were procured within the framework of the European Renal cDNA Bank–Kröner–Fresenius Biopsy Bank. Biopsies were obtained from patients after informed consent and with the approval of the local ethics committees [65]. Following a renal biopsy, the tissue was transferred to RNase inhibitor and microdissected into glomeruli and tubulointerstitium. Total RNA was isolated from micro-dissected glomeruli, reverse transcribed, and linearly amplified according to a protocol previously reported [66]. CEL file normalization was performed with the Robust Multichip Average method using RMAExpress (Version 1.0.5) and the human Entrez-Gene custom CDF annotation from Brain Array version 18 (http://brainarray.mbni.med.umich.edu/Brainarray/default.asp). To identify differentially expressed genes, the SAM (Significance Analysis of Microarrays) method was applied using TiGR (MeV, Version 4.8.1) [67]. Published gene expression profiles from patients with RPGN as well as controls (living donors (LD)) were used in this study (GSE104954, GSE104948,).

Statistical analysis: Data were expressed as mean ± SEM. Data from wild type and knockout mice were compared with one-way ANOVA on ranks, followed by the Student–Newman–Keuls test using SigmaStat Software (Jandel Scientific, Erkrath, Germany). The student *t*-test was used for direct comparisons between single groups—i.e., wild type and knockout cells/mice in case of normally distributed data or samples size *n* > 15. Mann–Whitney U test was used to analyze data with small sample size and non-parametric distribution of data. We used GraphPad Prism software. A *p*-value < 0.05 indicated statistical significance. Statistical significance was indicated as follows: *p*-value of <0.05 (*); *p*-value of <0.01 (**); *p*-value of <0.001 (***).

## Abbreviations

MDPI	Multidisciplinary Digital Publishing Institute
DOAJ	Directory of open access journals
TLA	Three letter acronym
LD	Linear dichroism
